# Soluble Urokinase Plasminogen Activator Receptor Is Associated With Subclinical Myocardial Impairment by Speckle Tracking Echocardiography in Lung Cancer Patients

**DOI:** 10.3389/fcvm.2021.659524

**Published:** 2022-01-28

**Authors:** Ahmad S. Manshad, Fatima A. Ballout, Jeffrey A. Borgia, Jochen Reiser, Tochukwu M. Okwuosa

**Affiliations:** ^1^Department of Internal Medicine, Rush University Medical Center, Chicago, IL, United States; ^2^Department of Anatomy and Cell Biology, Rush University Medical Center, Chicago, IL, United States; ^3^Department of Pathology, Rush University Medical Center, Chicago, IL, United States; ^4^Division of Cardiology, Rush University Medical Center, Chicago, IL, United States

**Keywords:** soluble urokinase plasminogen activator receptor (suPAR), myocardial global longitudinal strain, speckle tracking echocardiography, biomarkers, cancer

## Abstract

**Background:**

Plasma cardiac biomarkers have emerged as a cost-effective diagnostic tool aimed at early identification of cardiotoxicity. Soluble urokinase plasminogen activator receptor (suPAR) is a bone marrow cell derived signaling molecule that is associated with cardiovascular disease outcomes.

**Objectives:**

We investigated associations between suPAR and global longitudinal strain (GLS) as a marker of early myocardial impairment in lung cancer patients.

**Methods:**

We retrospectively analyzed 52 patients with stage IV non-small cell lung cancer with normal left ventricular ejection fraction (LVEF >55%) and without known heart disease or end-stage renal disease (ESRD). We studied associations between cardiac biomarkers and echocardiographic measures of systolic and diastolic function. GLS was analyzed using 2D speckle-tracking echocardiography via vendor-independent software (TomTec).

**Results:**

Median plasma suPAR was 7.0 ng/mL (interquartile range: 5.4–9.0). Mean LVEF was 61.9 ± 8.3% and mean GLS was-19.3 ± 2.1%. Inter-observer reproducibility was excellent for GLS as determined by Intraclass Correlation Coefficient analysis, ICC = 0.81 (0.68–0.89). After multivariate analysis, suPAR was the only biomarker associated with GLS (*p* = 0.009). suPAR was also associated with diastolic parameters E velocity (*p* = 0.018), A velocity (*p* = 0.017), and E/E' ratio (*p* = 0.033). Interestingly, suPAR was not associated with LVEF (*p* = 0.916). In addition, suPAR and GLS were found to be age-independent predictors of all-cause mortality, though only GLS remained significant after multivariate adjustment.

**Conclusions:**

In this cohort of stage IV non-small cell lung cancer patients with normal LVEF and without known heart disease or ESRD, suPAR was associated with GLS and diastolic impairment. suPAR is a readily available inexpensive biomarker; further research is required to evaluate the possible role of suPAR in screening for subclinical LV dysfunction in the high-risk oncological population.

## Introduction

Current standards for detecting cancer-therapy induced cardiotoxicity are based on assessment of cardiac function by left ventricular ejection fraction (LVEF) using either transthoracic echocardiography (TTE) or radionuclide multigated acquisition (MUGA) (1, 2). However, the assessment of LVEF lacks the sensitivity needed for detecting early subclinical changes. Newer echocardiographic modalities such as speckle tracking echocardiography (STE) enable earlier diagnosis of subclinical cardiac impairment not detected by conventional echocardiography (3).

Strain imaging, particularly global longitudinal strain (GLS) assessment by STE, has been increasingly utilized to risk stratify patients receiving anthracycline-based chemotherapeutic agents due to its superiority in detecting subclinical cardiac dysfunction (4, 5). STE by GLS therefore detects early derangements in cardiac function prior to a detectable fall in LVEF.

Biomarkers have emerged as a new cost-effective diagnostic tool aimed at early identification of patients more prone to developing cardiotoxicity (6). Soluble urokinase plasminogen activator receptor (suPAR) is gaining increasing attention because it is a circulating signaling molecule from the Ly6/neurotoxin family that is strongly predictive of incident and progressive chronic kidney disease and cancer cell progression (7–10). Mechanistically, suPAR activates podocytes on the kidney filtration barrier causing their functional breakdown (11) yet a mechanistic role for suPAR in cardiovascular diseases is not established. As an indicator of cardiovascular health, suPAR outperforms traditional markers of inflammation such as high sensitivity C-reactive protein (hs-CRP) in prognosticating a range of cardiovascular diseases (12, 13). Given the fundamental role of inflammation in cardiovascular disease (CVD), suPAR may aid in risk prediction and prevention of cardiac disease, particularly in the high-risk oncologic population.

Non-small cell lung cancer (NSCLC) accounts for the majority all lung cancers and is currently the leading cause of cancer-related deaths (14). Lung cancer patients are at increased risk of CVD due to direct cardiac toxicity from antineoplastic agents and radiation therapy, as well as shared cardiovascular risk factors (15). As new targeted therapies improve cancer patient survival, early detection of myocardial dysfunction is of utmost importance.

In the current study, we hypothesize that lung cancer treatment is associated with early/subclinical myocardial impairment as assessed by GLS. We examined the utility of an inexpensive and easily available biomarker (suPAR) in its association with GLS derangements as a marker of subclinical LV dysfunction, in stage IV non-small cell lung cancer patients with normal LVEF and without known heart disease or end-stage renal disease (ESRD).

## Methods

### Study Population

We selected patients with stage IV NSCLC that previously failed first-line platinum-based therapy and presented to Rush University Medical Center in Chicago, Illinois between January 2005 and December 2015. Serum and clinical data were collected prospectively by the Rush Biorepository Core (16) with written informed patient consent. The study protocol was reviewed and approved by the Institutional Review Board at RUMC.

**Figure 1:**
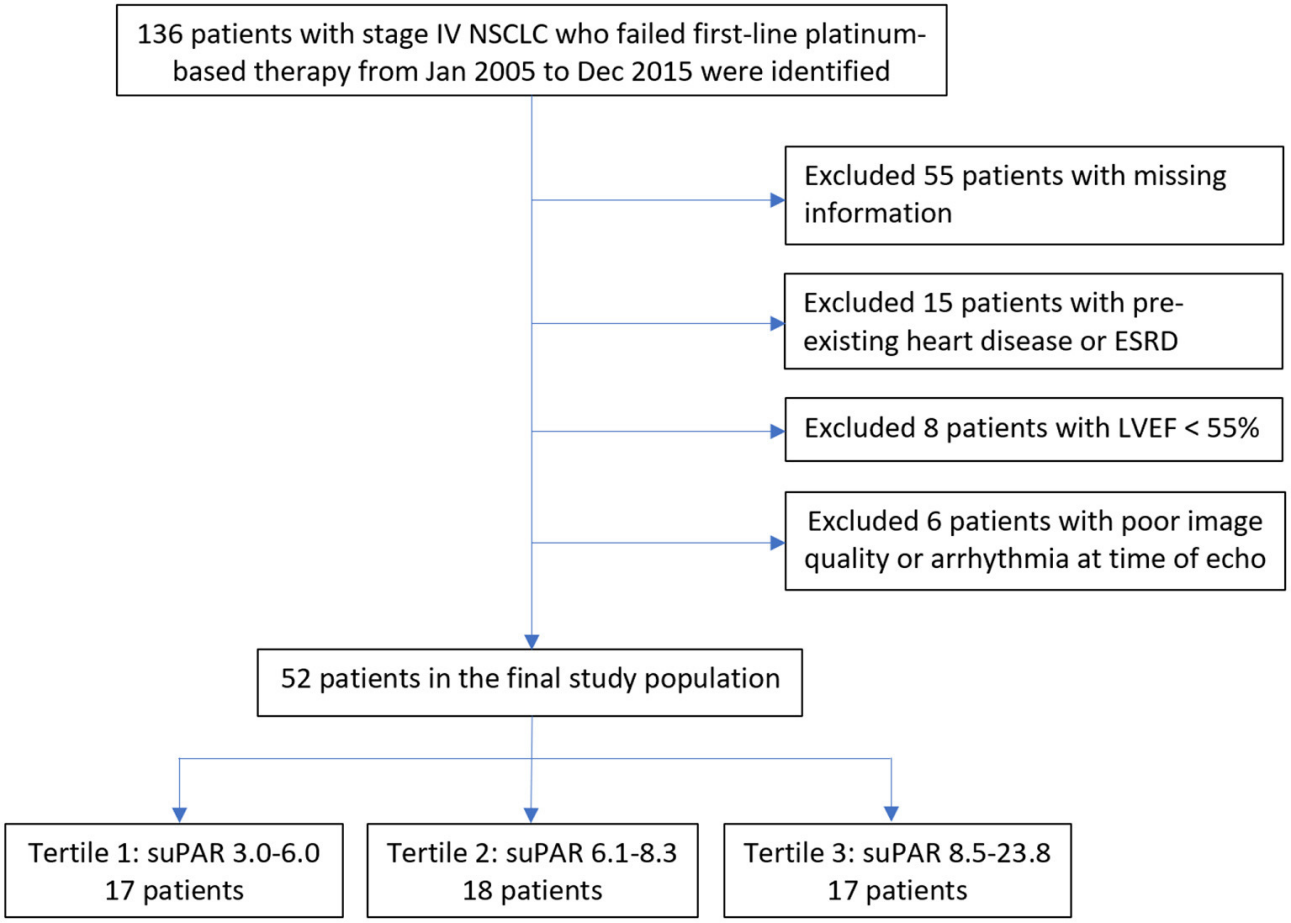
Study population flowchart. Displays the initial study population through the final study population, exclusions included. ESRD, End Stage Renal Disease; LVEF, Left Ventricular Ejection Fraction; NSCLC, Non-Small Cell Lung Cancer; suPAR, soluble urokinase-type Plasminogen Activator Receptor.

From a total of 136 patients with stage IV NSCLC whom had serum suPAR measurements available, we excluded patients with (1) incomplete data, (2) known heart disease and/or ESRD, (3) biplane LVEF < 55%, or (4) poor image quality or arrhythmia at the time of echocardiography (Figure 1). A total of 52 patients were included in the current study. ESRD was defined as estimated glomerular filtration rate <15 ml/min, based on Modification of Diet in Renal Disease method (17). Known heart disease was defined as heart failure; coronary artery disease, including previous myocardial infarction, stable angina, previous percutaneous coronary intervention or coronary artery bypass surgery; congenital heart disease; pacemaker or intracardiac defibrillator implantation.

### Outcome Measures

The time of observation was calculated from the date of suPAR draw until death or to the date of last follow-up for those still alive. All-cause mortality was obtained from the Social Security Death Index. Survival times were calculated from the date of suPAR draw to the date of death. Cox regression analysis was performed to predict 5-year all-cause mortality.

### Conventional Echocardiography

Comprehensive echocardiographic examinations were carried out and analyzed using General Electric, Vivid 7 Dimension imaging system device (GE Vingmed Ultrasound AS, Horten, Norway) with a 3.5 MHz transducer in accordance with the standard recommendations of the American Society of Echocardiography (18). Echocardiography was performed within 90 days of peripheral blood draw. LVEF was measured by biplane Simpson method in apical 4- and 2-chamber views. Three consecutive heart cycles were recorded for each view.

Pulsed-wave Doppler was performed in the apical 4-chamber view to obtain mitral inflow velocities for LV filling pattern evaluation. Peak velocity of early (E) and atrial (A) diastolic filling and deceleration time of E wave (DT) were measured, and the E/A ratio was calculated. Tissue Doppler early diastolic mitral annular velocity (E′) was acquired at the lateral annular site and used to calculate E/E' (19, 20). Diastolic function was classified as normal, mild (grade 1, impaired relaxation), moderate (grade 2, pseudonormal), or severe (grade 3, restrictive) (20).

### Speckle Tracking Echocardiography

**Figure 2:**
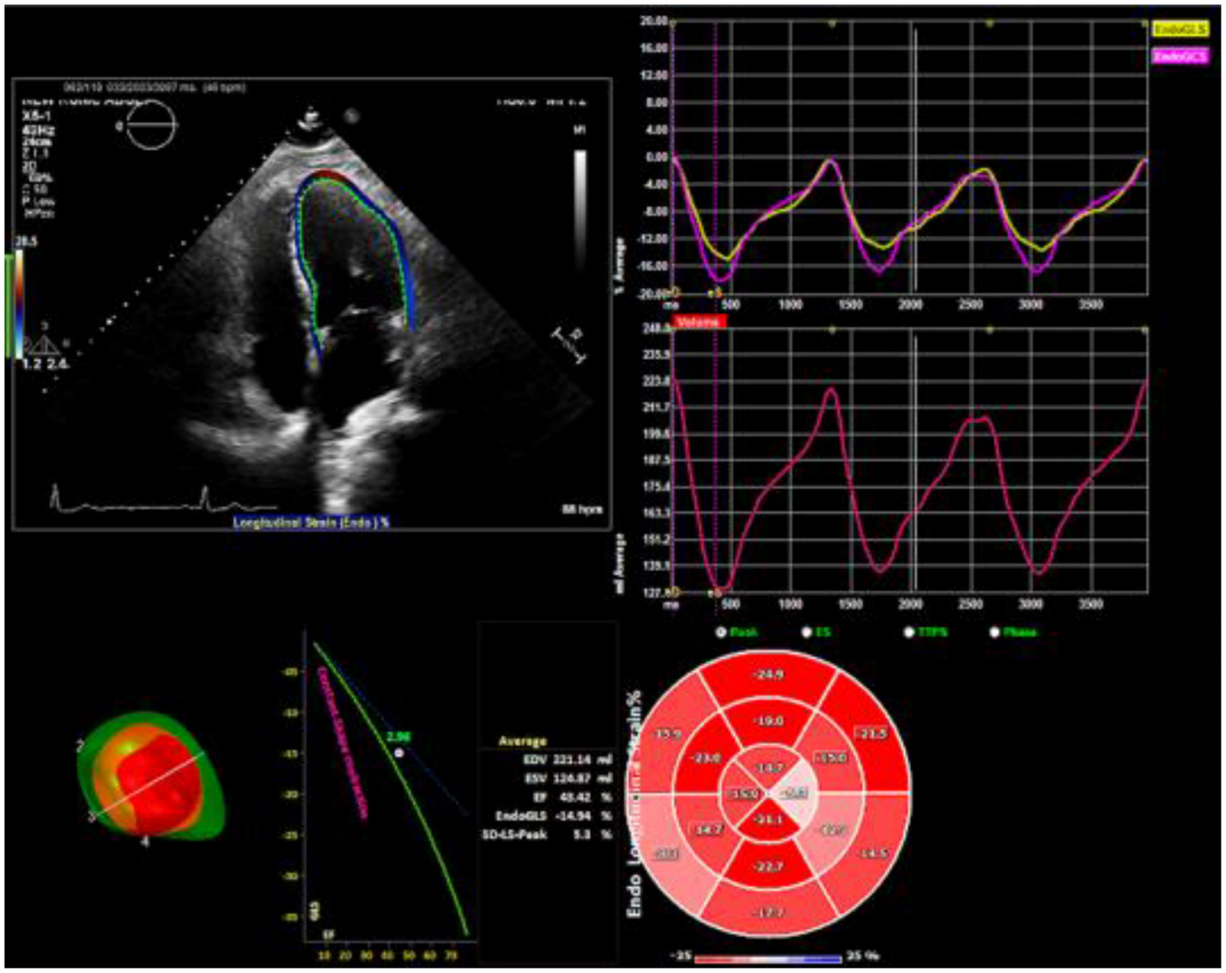
Two-dimensional speckle tracking echocardiography analysis. Strain curves and a color-coded 16-segment bull's eye plot are presented. Color lines indicate regional strain. Values of longitudinal strain are negative (sign –). Endocardial border tracing in apical four-chamber view can be achieved automatically. Global longitudinal strain (GLS) can be calculated from standard apical 4-, 2-, and 3- chamber views.

All echocardiographic images were acquired with frame rates of 70–90 frame/s and digitally stored for three cardiac cycles. This method has been described in detail (3) and involves tracking speckles from frame to frame. For the current study, the stored images were retrospectively assessed using 2D STE offline analysis software (2D Cardiac Performance Analysis) developed by TomTec Imaging Systems, GmbH (Munich, Germany). LV GLS was determined by selecting the most representative of the 3 cardiac cycles and marking the endocardium in the standard apical 4-, 2-, and 3- chamber views (Figure 2). Automated computation was then performed based on the timing of the aortic valve closure. Images were reviewed and analyzed offline by two independent observers blinded to suPAR levels and clinical characteristics of the study population.

### Laboratory Analysis

#### Collection and Storage of Serum Specimens

Peripheral blood was obtained from each patient using standard phlebotomy techniques, with all samples handled and processed in an identical manner, as previously described (21). A portion of each serum sample used for the Luminex evaluations were supplemented with 25 μL/mL of the Mammalian Protease inhibitor cocktail (Sigma, St. Louis, MO) and 10 μL/mL of 0.5 M EDTA to minimize further proteolysis. Aliquots were archived in a −80°C freezer until testing. No specimen was subjected to more than two freeze-thaw cycles.

#### Measurement of Serum Biomarker Concentrations

All specimens were evaluated using the Luminex immunobead platform and commercially-available kits, as previously described (21). All assays were performed according to the manufacturer's recommended protocols. All primary data points were collected on a Luminex FLEXMAP 3D® system with concentrations calculated based on 7-point standard curves using a five-parametric fit algorithm in xPONENT® v4.0.3 (Luminex Corp., Austin, TX) as previously described (21). sAXL and suPAR levels were measured using the commercially available MILLIPLEX® MAP Human Angiogenesis Panel 2 (EMD Millipore Corp., Billerica, MA) for Osteopontin levels. CRP levels were measured using Human Acute Phase 5+4-plex Panel (Bio-Rad Laboratories, Inc., Hercules, CA). Tumor necrosis factor-α (TNF-α) and interleukin-6 (IL-6) levels were measured using MILLIPLEX® MAP Human Circulating Cancer Biomarker Panel 1 (EMD Millipore Corp., Billerica, MA). Angiopoietin-2 and Endothelin-1 levels were measured using MILLIPLEX® MAP Human Angiogenesis/Growth Factor Panel 1 (EMD Millipore Corp., Billerica, MA). Resistin levels were measured using Human Diabetes 10-plex (Bio-Rad Laboratories, Inc., Hercules, CA). With a few exceptions, the blood samples were drawn within 6 months of cancer diagnosis; after the patients had failed platinum-based therapy.

### Statistical Analysis

For each patient the following data was obtained through electronic medical records: patient demographics, cancer history, cancer therapy, cardiac medication use, cardiac risk factors (e.g., hypertension, diabetes, hyperlipidemia, smoking), and eGFR. Continuous variables were reported as means ± SD while categorical variables were expressed as numbers or ratios. Between-group comparisons were achieved by 1-way ANOVA for continuous variables while chi-square test was used to evaluate dichotomous variables. Due to the skewed distribution of the biomarker levels, a natural logarithm transformation was performed on all biomarkers. The Pearson correlation coefficient was used to assess the correlation between two variables. A value of *p* < 0.05 was considered to be statistically significant. Reproducibility for LV GLS measurement was assessed by intraclass correlation coefficient (ICC) analysis.

Multivariable linear regression analysis was used to test associations between cardiac biomarkers and echocardiographic parameters. With fixed adjustments for age and sex, the forward stepwise selections approach (probability of 0.05 to enter or leave the model) was used to identify significant variables associated with systolic echocardiographic parameters (LVEF and GLS) and diastolic parameters (DT, E, A, E/A, E', and E/E'). Variables included in the final model were age, sex, BMI, smoking history, systolic blood pressure, diabetes, serum creatinine, and poorly differentiated histology. Potential risk factors for 5-year all-cause mortality were evaluated using Cox proportional hazards models. The multivariate model included age, gender, and additional variables; with a *p*-value of < 0.10 in the univariate Cox analysis. Variables included in the final model were age, gender, diabetes, use of diuretics, and use of beta blockers. All analyses were performed with SPSS version 22 for Windows (SPSS Inc., Chicago, IL, USA). Level of significance was set at *p* < 0.05.

## Results

### Patient Characteristics

Patient baseline data and echocardiographic parameters are summarized by suPAR tertiles in Table 1. The mean age was 62.7 ± 9.4 years, with 27 (52%) females. The median plasma suPAR level was 7.0 (interquartile range: 5.4–9.0). suPAR levels were similar in men and women, and were not associated with age or BMI. Higher suPAR levels were associated with history of smoking, use of diuretics, higher serum creatinine and lower eGFR, CRP, TNF-α, sAXL, and Angiopoietin-2. suPAR levels were not found to be associated with cancer duration, poorly differentiated histology, history of surgical resection, performance status, or radiation therapy.

**Table 1:** Baseline characteristics according to suPAR tertiles.

**Parameters**	**All patients (*n* = 52)**	**Tertile 1 (*n* = 17)**	**Tertile 2 (*n* = 18)**	**Tertile 3 (*n* = 17)**	***P*-value**
suPAR range, pg/mL	2,968–237,980	2,968–6,036	6,090–8,324	8,519–23,798	N/A
**Clinical characteristics**					
Age, years	62.7 ± 9.4	61.9 ± 9.5	60.9 ± 9.2	65.4 ± 8.2	0.308
Female, *n* (%)	27 (52%)	11 (65%)	11 (61%)	5 (29%)	0.077
African American, *n* (%)	10 (19%)	6 (35%)	2 (11%)	2 (12%)	0.127
Body mass index, kg/m^2^	25.0 ± 4.9	24.2 ± 4.8	25.4 ± 5.0	25.4 ± 4.9	0.741
Systolic BP, mmHg	121 ± 18	120 ± 12	118 ± 21	126 ± 19	0.385
Diastolic BP, mmHg	72 ± 12	69 ± 10	71 ± 12	76 ± 12	0.207
Smoking history, pack years	27 ± 29	15 ± 14	24 ± 22	44 ± 40	0.014
eGFR, mL/min	86 ± 28	109 ± 24	86 ± 24	62 ± 15	<0.001
**Medical history**					
Hypertension, *n* (%)	27 (52%)	7 (41%)	8 (44%)	12 (71%)	0.176
Dyslipidemia, *n* (%)	18 (35%)	6 (35%)	4 (22%)	8 (47%)	0.316
Diabetes, *n* (%)	7 (13%)	2 (12%)	3 (17%)	2 (12%)	0.892
**Cancer history**					
Cancer duration, months	14 ± 12	15 ± 14	10 ± 8	19 ± 13	0.098
Poorly differentiated, *n* (%)	27 (52%)	10 (59%)	10 (56%)	7 (41%)	0.563
Surgical resection, *n* (%)	27 (52%)	12 (71%)	7 (39%)	8 (47%)	0.159
Radiation therapy, *n* (%)	5 (10%)	1 (6%)	2 (11%)	2 (12%)	0.824
Performance status, grade	0.7 ± 0.6	0.6 ± 0.6	0.7 ± 0.6	0.9 ± 0.7	0.232
**Medications**					
Diuretics, *n* (%)	13 (25%)	0 (0%)	3 (17%)	10 (59%)	<0.001
Beta blockers, *n* (%)	24 (46%)	5 (29%)	10 (56%)	9 (53%)	0.249
Calcium channel blockers, *n* (%)	17 (33%)	5 (29%)	5 (28%)	7 (41%)	0.672
Ace inhibitors/ARBS, *n* (%)	15 (29%)	3 (18%)	4 (22%)	8 (47%)	0.129
Statins, *n* (%)	19 (37%)	8 (47%)	4 (22%)	7 (41%)	0.290
**Biomarkers**					
Creatinine, mg/dL	1.0 ± 0.4	0.7 ± 0.2	0.9 ± 0.3	1.2 ± 0.3	<0.001
CRP, mg/L	16.3 ± 17.6	6.8 ± 7.2	14.8 ± 11.7	27.4 ± 23.6	0.001
IL-6, ng/mL	6.6 ± 9.8	4.3 ± 7.8	4.6 ± 5.8	11.2 ± 13.4	0.065
TNF-α, ng/mL	6.0 ± 5.0	4.3 ± 3.5	5.2 ± 2.7	8.6 ± 7.0	0.030
sAXL, pg/mL	1372.1 ± 660.8	1098.8 ± 501.9	1193.2 ± 478.1	1843.8 ± 734.9	0.001
Angiopoietin-2, pg/mL	2,389 ± 1,679	1,590 ± 1,106	2,626 ± 1,951	2,937 ± 1,630	0.046
Resistin	4,982 ± 2,704	4,106 ± 1,891	5,081 ± 2,442	5,755 ± 3,455	0.205
Endothelin-1, pg/mL	18.0 ± 77.3	37.3 ± 135.5	7.4 ± 4.4	10.0 ± 7.0	0.462
**Echocardiographic characteristics**					
LVEF, %	61.9 ± 8.3	61.7 ± 8.0	62.9 ± 10.0	61.1 ± 6.8	0.805
GLS, %	−19.3 ± 2.1	−20.3 ± 1.9	−19.6 ± 1.6	−17.8 ± 2.1	0.001
E velocity, m/s	0.8 ± 0.2	0.7 ± 0.1	0.8 ± 0.1	0.9 ± 0.2	0.034
A velocity, m/s	0.8 ± 0.2	0.8 ± 0.2	0.8 ± 0.2	0.9 ± 0.2	0.107
E/A ratio	1.0 ± 0.3	1.0 ± 0.3	1.0 ± 0.2	1.0 ± 0.3	0.881
E' velocity, cm/s	9.2 ± 1.7	9.5 ± 1.3	9.0 ± 1.4	9.2 ± 1.2	0.698
E/E' ratio	8.8 ± 2.4	7.6 ± 1.5	8.9 ± 1.9	9.9 ± 3.2	0.025
Deceleration time, ms	195 ± 75	202 ± 45	187 ± 56	198 ± 112	0.828

*P–values are for unadjusted comparisons (analysis of variance or χ^2^) between tertiles of suPAR. Values are presented as mean ± standard deviation or as percentages (%). BP, blood pressure; CRP, C–reactive protein; GFR, glomerular filtration rate; IL−6, interleukin-6; LVEF, left ventricular ejection fraction; sAXL, soluble AXL; TNF-α, tumor necrosis factor-α*.

### suPAR and Echocardiographic Parameters

**Figure 3:**
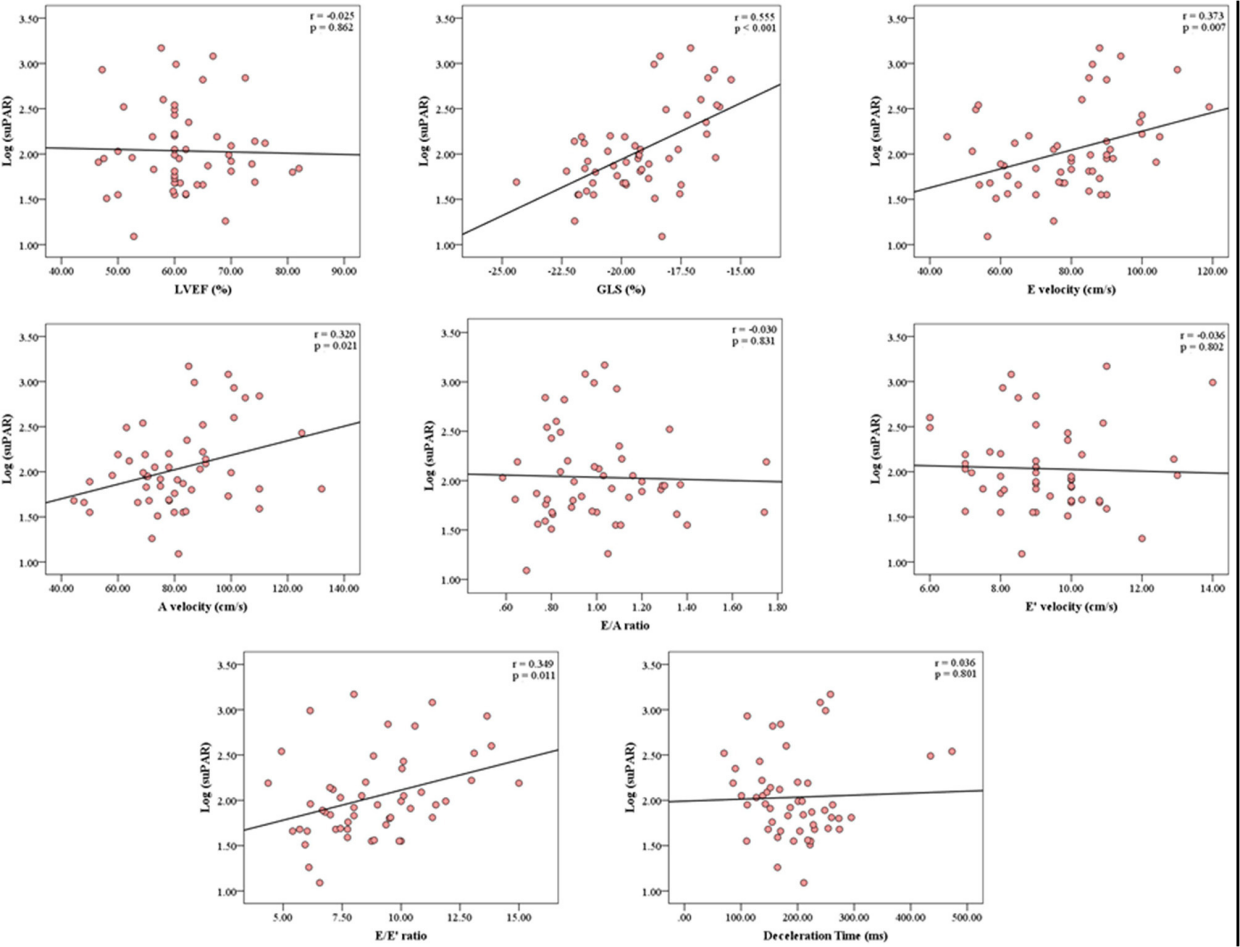
Correlation Analysis between the serum concentration of suPAR and echocardiographic parameters. The correlation analysis graphs demonstrate significant correlations between suPAR levels and GLS, E velocity, A velocity, and E/E'. suPAR levels were not associated with LVEF. GLS, Global Longitudinal Strain; LVEF, Left Ventricular Ejection Fraction; r, Pearson's correlation coefficient; suPAR, soluble urokinase-type Plasminogen Activator Receptor.

The mean LVEF was 61.9 ± 8.3% and mean GLS was −19.3 ± 2.1%. Inter-observer reproducibility was excellent for GLS, ICC = 0.81 (0.68–0.89). suPAR levels were not associated with LVEF (*p* = 0.862) in unadjusted comparisons (Figure 3). Conversely, there was a significant association between suPAR and GLS (*p* < 0.001), which remained statistically significant (*p* = 0.009) in the multivariate adjusted model (Table 2). For the echocardiographic parameters, suPAR levels were significantly correlated with diastolic measures E velocity (*p* = 0.007), A velocity (*p* = 0.021), and E/E' (*p* = 0.011), but not with E/A (*p* = 0.831), E' (*p* = 0.802), or DT (*p* = 0.801) in unadjusted comparisons; but only E velocity, A velocity, and E/E' remained significantly associated with suPAR (*p* = 0.018, *p* = 0.017, and *p* = 0.033, respectively) in multivariate analysis.

**Table 2:** Multivariate linear regression analysis showing association between suPAR and echocardiographic parameters after adjusting for important covariates.

	**β (95% CI)**	***P*-value**
LVEF (%)	0.334 (−6.025 to 6.693)	0.916
GLS (%)	1.710 (0.446–2.975)	0.009
E velocity	14.845 (2.694–26.995)	0.018
A velocity	17.097 (3.193–31.002)	0.017
E/A ratio	−0.057 (−0.268 to 0.154)	0.590
E' velocity	−0.158 (−1.229 to 0.913)	0.767
E/E' ratio	1.721 (0.142–3.301)	0.033
DT	13.625 (−45.388 to 72.637)	0.644

*Model adjusts for age, sex, BMI, smoking history, SBP, diabetes, creatinine, and poorly differentiated histology. DT, Deceleration Time; LVEF, Left Ventricular Ejection Fraction; GLS, Global Longitudinal Strain*.

### suPAR, Cardiac Biomarkers, and GLS

Associations between suPAR, cardiac biomarkers, and GLS were examined in unadjusted and adjusted models (Table 3). In unadjusted comparisons, suPAR (*p* < 0.001), TNF-α (*p* = 0.032), and sAXL (*p* = 0.033) were significantly correlated with GLS. However, after multivariate adjustment, only suPAR (*p* = 0.009) remained associated with GLS.

**Table 3:** Univariate and Multivariate associations between biomarkers and GLS.

**Biomarker**	**Univariate**		**Multivariate**	
	***r*-value**	***P*-value**	**β (95% CI)**	***P*-value**
suPAR	0.555	<0.001	1.710 (0.446–2.975)	0.009
IL−6	0.122	0.398	0.114 (−0.138 to 0.367)	0.366
TNF-α	0.297	0.032	0.184 (−0.402 to 0.770)	0.532
CRP	0.095	0.504	0.135 (−0.301 to 0.571)	0.536
sAXL	0.295	0.033	0.287 (−0.901 to 1.475)	0.628
Angiopoietin-2	0.117	0.410	0.035 (−0.811 to 0.882)	0.933
Resistin	0.159	0.259	0.001 (−1.163 to 1.164)	0.999
Endothelin−1	0.101	0.478	−0.154 (−0.830 to 0.521)	0.647

*Model adjusts for age, sex, BMI, smoking history, SBP, diabetes, creatinine, and poorly differentiated histology. CRP, C-reactive protein; IL-6, interleukin-6; LVEF, Left Ventricular Ejection Fraction; GLS, Global Longitudinal Strain; r-value, Pearson's correlation coefficient; sAXL, soluble AXL; TNF-α, tumor necrosis factor-α*.

### Prognostic Value of suPAR and GLS

The median follow-up time was 7.5 months (interquartile range: 3.75–18). At 5 years, 51 patients (98%) were deceased. In univariate Cox regression, the use of diuretics and beta blockers were the only clinical factors associated with all-cause mortality. In unadjusted models, GLS and multiple biomarkers including suPAR, CRP, TNF-α, angiopoietin-2, and resistin were independently associated with all-cause mortality (data not shown). After adjusting for age, suPAR, and GLS remained significantly associated with all-cause mortality. However, in the multivariate model, only GLS remained an independent predictor of all-cause mortality (data not shown).

## Discussion

In this study of 52 pretreated stage IV NSCLC patients with normal LVEF and without ESRD, we investigated associations between the biomarker suPAR and GLS as a measure of subclinical LV dysfunction. We also assessed associations between suPAR, other biomarkers, and echocardiographic measures of systolic and diastolic function.

We demonstrated that suPAR was strongly and independently associated with GLS; but not LVEF. In comparison to other cardiac biomarkers, suPAR was the only biomarker associated with GLS after multivariate adjustment. Furthermore, both suPAR and GLS were found to be independent predictors of all-cause mortality, independent of age. These findings suggest that suPAR is a sensitive marker of early myocardial impairment with useful prognostic implications.

CVD is an important cause of morbidity and mortality in the oncological population due to shared cardiovascular risk factors and direct cardiotoxic effects of cancer therapy. However, current standards for monitoring cardiac dysfunction rely on the presence of functional impairment, precluding any chance of preventing its development (1, 2). In the case of anthracycline-associated LVEF impairment, early initiation (within the first month of discovery of LVEF impairment) of standard heart failure therapy was associated with two-thirds chance of full LVEF recovery, compared with a 0% chance of full recovery if treatment was initiated after 6 months (22). Therefore, more sensitive screening modalities are needed for earlier detection of subclinical heart disease and stratification of patients prone to developing myocardial impairment. Several population-based studies have demonstrated a link between suPAR and CVD and mortality in the general population (9). Our study is the first to investigate associations between plasma suPAR levels and subclinical myocardial impairment in the oncological population.

GLS assessed by STE is an emerging technique for detecting and quantifying subclinical LV systolic dysfunction. GLS is established to be the best measure for predicting cardiotoxicity and clinical dysfunction in cancer patients receiving chemotherapy (4). Furthermore, GLS was found to be superior to LVEF in predicting cardiac events and all-cause mortality in patients with previous CVD or chronic kidney disease (5, 23). However, GLS is not routinely used in practice due to lack of standardization across echocardiographic imaging software and hardware; and the relatively time-consuming nature of GLS acquisition with echocardiographic imaging (24, 25); which is also not currently reimbursed in the United States.

Over the last decade, measurement of cardiac-specific biomarkers has emerged as a new cost-effective diagnostic tool aimed at early identification of patients more prone to developing cardiotoxicity (6). suPAR is thought to reflect activation of the inflammatory and immune systems and has been associated with poor clinical outcomes (12, 26, 27). suPAR has been associated with the presence of coronary micro- and macrovascular disease, carotid plaques, stroke, myocardial ischemia, and cardiovascular death, independent of traditional CV risk factors and hs-CRP (9, 13, 28–30). Moreover, suPAR, which has been linked to vascular inflammation, is a better marker for CVD compared with other markers of inflammation such as hs-CRP (12, 13, 31, 32). Despite the observed association between suPAR and several aspects of CVD, it remains unclear whether suPAR is playing a causal role.

In our cohort of stage IV NSCLC patients, the levels of suPAR were noticeably greater than those in similar non-oncological study populations. This is in line with previous studies in cancer patients showing more significant suPAR elevations when compared with healthy controls (9, 33). Urokinase-type plasminogen activator receptor (uPAR) is present in NSCLC tissue (7) and is thought to be released into the plasma leading to increased suPAR levels. Our patient cohort was treated with antineoplastic drugs, including first-line platinum-based therapy, at the time of the study. Therefore, it cannot be determined from the present data whether this treatment may have caused the release of suPAR (e.g., from dead cancer cells) or whether the plasma suPAR levels are independent of antineoplastic treatment. Notably, platinum-based therapy has not been shown to be related to diastology (15) or general LV dysfunction, including LVEF (15, 34). We demonstrated that suPAR levels were not significantly associated with history of radiation therapy, surgical resection, poorly differentiated histology, or cancer duration. We suspect this may be related to the variability of timing of symptom onset to diagnosis in these late stage cancer patients.

In the current study, we examined the relationship between cardiac biomarkers and systolic function in cancer patients with normal LVEF and without ESRD. We demonstrated that suPAR was the only biomarker associated with GLS after multivariate adjustment. Interestingly, we found no association between suPAR and LVEF, suggesting that suPAR may be more reflective of early myocardial changes. These findings are supported by Theidela et al. in a diabetic population with normal LVEF and without ESRD (35). In contrast, Fujita et al. showed an association between suPAR and LVEF in patients with ischemic heart disease and impaired renal function (36), likely because suPAR levels are elevated in patients with renal disease (10) as was the case in our univariate analysis.

We also examined the relationship between suPAR and diastolic parameters. Consistent with other studies (35, 37) suPAR was significantly associated with diastolic function (E/E') after multivariate adjustment. This could be related to impaired coronary microcirculation and increased arterial stiffness seen in patients with elevated suPAR levels (37–40).

It is noteworthy that in the early results of the Strain Surveillance of Chemotherapy for Improving Cardiovascular Outcomes [SUCCOR] randomized trial (41), there was no significant difference between GLS and LVEF-guided management of potential cardiotoxicity in majority breast cancer patients treated with doxorubicin therapy. Major critiques and flaws of this study were that first, unlike our current study, the mean dose of doxorubicin administered in the SUCCOR trial was <250 mg/m^2^, the threshold for cardiotoxicity risk associated with doxorubicin therapy. Second, LVEF and GLS in the SUCCOR trial were in the normal range at baseline and follow-up, with therefore limited power for detection of changes in LVEF or GLS. Third, the first two problems listed above were compounded by the short trial duration of follow-up, which further limited the ability of the study to detect differences between the two groups. Despite these challenges, the SUCCOR trial still demonstrated that fewer patients had cardiotoxicity in the GLS-guided than the LVEF-guided arm; and that among those that received medical therapy for cardiotoxicity risk, there were larger reductions in LVEF at follow-up in the LVEF-guided arm compared with the GLS-guided arm.

The prognostic value of both suPAR and CRP have been well-documented in different types of cancers, including NSCLC (35, 41, 42). Similarly, GLS is a useful prognostic marker in multiple disease processes, including CVD and malignancy (9). In line with previous studies, we demonstrate consistent prognostic value of suPAR, CRP, and GLS in predicting all-cause mortality in our age-adjusted NSCLC cohort. However, after multivariate only GLS and CRP remain independent predictors of all-cause mortality.

Our results suggest that suPAR may be a valid biomarker for subclinical myocardial impairment as the association between suPAR and GLS remained significant even after adjustment of important covariates. Given this observed relationship between suPAR and subclinical myocardial dysfunction, suPAR measurements may therefore be useful in clinical practice in identifying oncological patients at risk of developing heart disease. suPAR as a simple, inexpensive, and readily available test, could be a useful surrogate marker to circumvent the difficulty and costs associated with serial GLS measurements, particularly in cancer survivorship years.

### Strengths and Limitations

As a single-center retrospective study, our study cannot provide information on the causal or resultant nature of the relationship between suPAR and early myocardial impairment. Furthermore, as a retrospective study, prior heart disease was determined by the information documented in the electronic medical records, and could not be ascertained. Echocardiographic examination and blood draw for suPAR measurements were not performed simultaneously, which could affect accurate comparisons. Also, because many of the patients were lost to follow-up in the community after cancer therapy, we did not have direct access to cardiovascular events data. We had access to the deaths data because of the cancer registry that meticulously obtains and records death information from the social security death index. Lastly, our small sample size was partly due to missing values which could have induced bias, thus limiting the interpretation of the results. Nonetheless, the robust associations between suPAR and GLS, despite the small sample size, emphasizes the strength of our study findings.

## Conclusions

In patients with stage IV NSCLC with normal LVEF and without known heart disease or end-stage renal disease, suPAR was significantly associated with GLS and markers of diastolic LV myocardial impairment. Additionally, suPAR outperformed other cardiac biomarkers in its association with GLS. Moreover, suPAR and GLS were found to be independent predictors of all-cause mortality, independent of age. suPAR is a readily available and inexpensive marker. In order to limit costs associated with serial echocardiographic imaging, further research is required to evaluate the possible role of suPAR in screening for subclinical LV dysfunction—during treatment and particularly in cancer survivorship years—in the high-risk oncological population.

## Data Availability Statement

The original contributions presented in the study are included in the article/supplementary material, further inquiries can be directed to the corresponding author/s.

## Ethics Statement

The studies involving human participants were reviewed and approved by Rush University Medical Center. Written informed consent for participation was not required for this study in accordance with the national legislation and the institutional requirements.

## Author Contributions

AM, FB, and TO designed and executed the study. JB performed biomarker collection. AM and TO analyzed the data. AM and FB drafted the initial manuscript. TO, JB, and JR revised and discussed the manuscript. All co-authors have contributed to the development of this research project and the writing of this manuscript. All authors read and approved the manuscript for publication.

## Conflict of Interest

JR is a cofounder of TRISAQ which develops drugs against suPAR and in which he has financial interest including stock. The remaining authors declare that the research was conducted in the absence of any commercial or financial relationships that could be construed as a potential conflict of interest.

## Publisher's Note

All claims expressed in this article are solely those of the authors and do not necessarily represent those of their affiliated organizations, or those of the publisher, the editors and the reviewers. Any product that may be evaluated in this article, or claim that may be made by its manufacturer, is not guaranteed or endorsed by the publisher.

## Abbreviations

CVD: cardiovascular disease
GLS: global longitudinal strain
TTE: transthoracic echocardiography
MUGA: radionuclide multigated acquisition
STE: speckle tracking echocardiography
LVEF: left ventricular ejection fraction
suPAR: soluble urokinase plasminogen activator receptor
CRP: C-reactive protein
NSCLC: non-small cell lung cancer
ESRD: end stage renal disease.
